# Acute Effects of the New Method Sarcoplasma Stimulating Training Versus Traditional Resistance Training on Total Training Volume, Lactate and Muscle Thickness

**DOI:** 10.3389/fphys.2019.00579

**Published:** 2019-05-15

**Authors:** Fernando Noronha de Almeida, Charles Ricardo Lopes, Raphael Machado da Conceição, Luan Oenning, Alex Harley Crisp, Nuno Manuel Frade de Sousa, Thiago Barbosa Trindade, Jeffrey M. Willardson, Jonato Prestes

**Affiliations:** ^1^Graduation Program on Physical Education, Catholic University of Brasilia, Brazilia, Brazil; ^2^Human Performance Research Group, Methodist University of Piracicaba, São Paulo, Brazil; ^3^Adventist Faculty of Hortolândia (UNASP), São Paulo, Brazil; ^4^Laboratory of Exercise Physiology, Faculty Estacio of Vitoria, Espírito Santo, Brazil; ^5^Department of Health and Human Performance, Montana State University Billings, Billings, MT, United States

**Keywords:** training method, muscle pump, training volume, resistance exercise, muscle thickness

## Abstract

**Background:** Trained subjects have difficulty in achieving continued results following years of training, and the manipulation of training variables through advanced resistance training (RT) methods is widely recommended to break through plateaus.

**Objective:** The purpose of the present study was to compare the acute effects of traditional RT (TRT) versus two types of sarcoplasma stimulating training (SST) methods on total training volume (TTV), lactate, and muscle thickness (MT).

**Methods:** Twelve trained males (20.75 ± 2.3 years; 1.76 ± 0.14 meters; body mass = 79.41 ± 4.6 kg; RT experience = 4.1 ± 1.8 years) completed three RT protocols in a randomly sequenced order: TRT, SST contraction type (SST-CT), or SST rest interval variable (SST-RIV) with 7 days between trials in arm curl (elbow flexors) and triceps pulley extension (elbow extensors) performed on the same day.

**Results:** The SST groups displayed greater acute biceps and triceps brachii (TB) MT versus the TRT session, with no difference in lactate levels between them. The SST-CT resulted in greater biceps and TB MT versus the SST-RIV session. The TTV was greater for the TRT session versus the SST sessions, except in the case of the elbow flexors (no difference was observed between TRT and SST-CT), and higher for the SST-CT versus the SST-RIV.

**Conclusion:** Trained subjects may benefit from using the SST method as this method may offer a superior MT stimulus and reduced training time, even with a lower TTV.

## Introduction

Resistance training (RT) is a popular exercise modality, and has been shown to increase strength (Prestes et al., 2015), muscle power (Hanson et al., 2009), functional capacity, muscle endurance, cardiovascular fitness, quality of life, and muscle hypertrophy (American College of Sports Medicine, 2009). The principle of diminishing returns applies to RT, in that trained subjects have difficulty in achieving continued results following years of training (Prestes et al., 2017). Thus, the manipulation of training variables through advanced resistance training methods is widely recommended to break through plateaus (Deschenes and Kraemer, 2002).

Some RT methods are designed to increase the time under tension and total training volume (TTV), which are important variables to be considered in training sessions designed to generate hypertrophy gains and metabolic stress (Schoenfeld et al., 2017). Burd et al. (2010) demonstrated that time under tension was a key variable to stimulate increases in acute protein synthesis that contributes to complex physiological mechanisms ultimately responsible for muscle hypertrophy. Nevertheless, muscle hypertrophy depends on shifting the muscle protein balance to favor synthesis over degradation (Schoenfeld, 2013). In this sense, training methods are also used to increase metabolic stress (local and circulating indicators, such as lactate) and time under tension through the manipulation of training volume, intensity, rest intervals between sets and exercises, muscle contraction type, and velocity (Schoenfeld et al., 2017). Moreover, metabolic stress induced by RT involves an increase in intracellular hydration and the raising of water content of the muscle cells, which has been suggested as an important stimulus for muscle growth in a condition of higher metabolic accumulation (Loenneke et al., 2012; Schoenfeld, 2013). There is also an association of acute muscle pump with the activation of integrin, a membrane protein responsible for the triggering of intracellular anabolic mechanisms and the reduction of catabolic processes (protein degradation), accompanied by increased muscle protein synthesis (Wackerhage et al., 2019). During the first 5 weeks of RT the increase in vastus lateralis cross-sectional area was associated with edema–induced muscle swelling as measured by ultrasound images, which was not elevated after 10 weeks, despite the increase in muscle cross-sectional area (Damas et al., 2016).

Nevertheless, Marshall et al. (2012) compared the acute effect of rest-pause versus traditional RT sessions in trained subjects that completed three different protocols for the squat exercise with 80% of one-repetition maximum. The protocols consisted of 5 sets of 4 repetitions with 3 min inter-set rest intervals; 5 sets of 4 repetitions with 20 s inter-set rest intervals; and the rest-pause method with a set to failure, with subsequent sets performed with a 20 s inter-set rest interval totalizing 20 repetitions. There was greater muscle electromyographic activity and no statistical difference in fatigue behavior during the rest-pause versus the traditional protocols, reinforcing the importance of RT methods to overcome plateaus. Moreover, trained subjects displayed higher energy expenditure and oxygen consumption for up to 22 h following the rest-pause method in the leg press, bench press, and lat pull-down compared with traditional RT (Paoli et al., 2012). Thus, the use of advanced RT methods such as rest-pause is an interesting strategy for trained subjects, which may also promote chronic adaptations (Prestes et al., 2017).

However, the comparison between advanced RT methods is scarce in literature, especially with regard to the most recent training approaches used in practice by bodybuilders and trained subjects; for example, the sarcoplasma stimulating training (SST) method.

The SST method is growing in popularity in the bodybuilding population and involves different types of muscle actions, and also includes very short rest intervals between sets to increase time under tension. The SST was originally developed by the Swiss coach Patrick Tuor to intensify RT sessions in highly trained athletes (Prestes et al., 2016). Tuor hypothesized that highly trained athletes would reach a point at which classic RT methods would no longer be effective due to high training tolerance; thus, their muscle cells would require a very distinct stimuli to be exhausted, and to adapt. Briefly, the idea is to start a set with 70–80% of one-repetition maximum to failure and repeat this procedure two more times with 20 s rest intervals. The next step is to remove 20% of the load and perform a set with 1 s of concentric phase and 4 s of eccentric phase; 20 s later, 20% of the load is removed again, and a set with 4 s of concentric phase and 1 s of eccentric phase is completed to failure. The last procedure is to remove 20% of the load and 20 s later perform an isometric muscle action (static hold) to failure. Another SST variation consists of using 70–80% of one-repetition maximum load to failure accompanied by programmed variable rest intervals between sets without reducing the load, somewhat similar to the rest-pause method, except for the variable rest interval, as follows: 45, 30, 15, 5, 5, 15, 30, and 45 s, totalizing eight sets. Considering the dearth of scientific data about the types of SST, some comparisons with similar training approaches will be necessary to improve the theoretical basis of its application (Prestes et al., 2017). During SST sessions training duration can widely vary due to metabolic stress (short rest intervals), while the main idea is to maintain the muscle as much as possible under tension, even with a reduced training duration (Prestes et al., 2016).

Thus, the aim of the present study was to compare the acute effects of traditional RT versus two types of the SST method on TTV, lactate, and muscle thickness (MT). Our hypothesis was that the SST method would result in greater acute MT, lactate levels, and lower TTV as compared to traditional RT, based on the prolonged local stress, and high fatigue induced by this method.

## Materials and Methods

### Subjects

Twelve trained males (20.75 ± 2.3 years; 1.76 ± 0.14 meters; total body mass = 79.41 ± 4.6 kg; RT experience = 4.1 ± 1.8 years; frequency = 4.5 ± 0.7 session ⋅ wk-1) volunteered to participate in this study. The sample size was justified by *a priori* power analysis based on a pilot study where the biceps brachii (BB) MT was assessed as the outcome measure with a target large effect size of 0.40 (using ANCOVA, main effects and interaction), an alpha level of 0.05, and a power (1-β) of 0.80. The sample size was determined using G^∗^Power version 3.1.3 (Faul et al., 2007). As inclusion criteria, subjects should be regularly performing all exercises utilized in the training intervention with a minimum frequency of once a week before entering the study, and should be performing RT a minimum of 3 days/week for at least 1 year at the University training facility. The range of RT experience was 2–8 years. Subjects with any existing musculoskeletal disorders, a history of injury with residual symptoms (pain, “giving-way” sensations) in the trunk, upper and lower limbs within the last year, and subjects who were taking anabolic steroids or any other illegal pharmacological agents known to increase muscle size at the time of selection and during the previous year, were excluded. This study was approved by the university research ethics committee (protocol 1.792.429); all subjects read and signed an informed consent document.

### Ten Repetitions Maximum Tests (10RM)

The 10RM tests were performed to determine the exact training load for standing arm curl (elbow flexors), and triceps pulley extension (elbow extensors). During the standing arm curl, subjects were advised to start with their elbows fully extended and to then flex their elbows as much as possible. For the triceps pulley extension, the elbows were positioned at a 90° angle and subjects were asked to fully extend their elbows. Ten minutes were allowed between the tests to avoid fatigue. The tests followed these procedures: warm-up on each exercise with 5–10 submaximal repetitions using a light load (60% of the estimated 10RM); 1-min rest, and load increments of 5–10% until the 10RM was found within 3–5 attempts, using 3- to 5-min rest intervals; subjects were instructed to lift and lower the load at a controlled velocity, approximately 2 s for each phase of the movement; 10 repetitions were recorded, with the maximal load determined by the last successful set of repetitions. Subjects were familiarized with both exercises in their training routines, and standardized instructions were provided. Consistent verbal encouragement was provided during the testing procedures to all subjects. All testing and training sessions were scheduled 7:00 PM in controlled room temperature.

### Resistance Training Sessions

Subjects completed three RT protocols in a randomly sequenced order, as follows: traditional resistance training (TRT), sarcoplasma stimulating training contraction type (SST-CT), or sarcoplasma stimulating training rest interval variable (SST-RIV), with 7 days between trials. Furthermore, they were advised to refrain from any type of regular exercise training in the days between testing sessions. The chosen exercises were standing arm curl (elbow flexors), and triceps pulley extension (elbow extensors) performed on the same day. Ten minutes of rest were allowed after the standing arm curl exercise before starting the triceps pulley extension exercise. The TRT session consisted of eight sets to failure (inability to complete a full concentric repetition with standardized movement technique) with a 10RM load and with 1 min rest intervals between them. The SST-CT session was as follows: an initial set with a 10RM load to failure, followed by two more sets with 20 s rest intervals while maintaining the load. After this, 20% of the load was removed, and subjects performed another set with repetitions that consisted of a 1 s concentric phase and a 4 s eccentric phase to failure; after another 20 s, 20% of the load was removed again, and an additional set was performed, consisting of repetitions with a 4 s concentric phase and a 1 s eccentric phase to failure. Finally, for the last set, 20 s later, an additional 20% of the load was removed and an isometric muscle action (static hold at 90° of elbow flexion) was held until failure, totalizing six sets. The SST-RIV consisted of an initial set with 10RM load to failure followed by programmed variable rest intervals between sets to failure without load reduction as follows: 45, 30, 15, 5, 5, 15, 30, and 45 s, totalizing eight sets.

### Total Training Volume (TTV)

Total training volume in arbitrary units (A.U.) for the standing biceps curl (elbow flexors), and triceps pulley extension (elbow extensors) was calculated by multiplying the total number of sets, the total number of repetitions, and the load used (kg) (American College of Sports Medicine, 2009).

### Analysis of Blood Lactate

All samples were obtained while subjects were seated. The right index finger was cleaned using alcohol prior to each blood draw; the first blood drop was discharged to avoid contamination of the sample. Blood samples of 25 μL were collected in heparinized capillary tubes and transferred to microtubes containing 50 μL of sodium fluoride at 1%. All samples were collected at the following time-points: pre-test, immediately post, 5-min, and 10-min post each protocol. Lactate concentration was analyzed via an electro enzymatic method with a lactate analyzer (YSI 2300 Stat Analyzer; Yellow Springs Instruments, Yellow Springs, OH, United States) previously calibrated, with results expressed in mmol/l. Of note was the fact that values from immediately post, 5 min, and 10 min post were not statistically different, and they were collectively presented only as post-training. Values were also analyzed in duplicate.

### Analysis of Muscle Thickness (MT)

Ultrasound imaging was used to obtain measurements of MT and was performed by a trained technician using an A-mode ultrasound imaging unit (Bodymetrix Pro System; Intelametrix Inc., Livermore, CA, United States). Following the application of a water-soluble transmission gel (Mercur S.A. – Body Care, Santa Cruz do Sul, Brazil) to the measurement site, a 2.5-MHz linear probe was positioned perpendicularly to the tissue interface without depressing the skin. The equipment settings were optimized to obtain the best quality images according to the manufacturer’s user manual and were held at a constant across testing sessions. When the quality of the image was deemed to be satisfactory, the image was saved to the hard drive, and MT dimensions were determined by measuring the distance from the subcutaneous adipose tissue–muscle interface to the muscle-bone interface. Measurements were taken on the right side of the body for the triceps brachii (TB) and biceps brachii (BB). Upper arm measurements were conducted while subjects were standing.

For the anterior and posterior upper arm, measurements were taken at 60% distal between the lateral epicondyle of the humerus, and the acromion process of the scapula. For each measurement, the examined limb was held constant in the same position to avoid movement. To maintain consistency between pre- and post-intervention testing, each site was marked with henna ink. To further ensure the accuracy of measurements, at least three images were obtained for each site. If measurements were within 1 mm of one another, the values were averaged to obtain a final value. If measurements were more than 1 mm of one another, a fourth image was obtained, and the closest three measurements were averaged. The test-retest intraclass correlation coefficient for TB and BB were 0.998 and 0.996, respectively, while the coefficient of variation was 0.6 and 0.4%, respectively. The standard error of the mean for these measures was 0.42 and 0.29 mm, respectively.

### Statistical Analysis

The data are expressed as mean and standard deviation (SD) values. The Shapiro–Wilk test was applied to check for the normality distribution of the study variables. ANCOVA was used to determine the effect of three different exercise-training systems on post-intervention MT and blood lactate concentration after controlling for pre-intervention variables. One-way repeated measures ANOVA was also used to determine the differences between interventions for TTV. Tukey’s *post hoc* test with the Bonferroni correction was applied in the event of significance. The effect size calculation (ES = difference between pre- and post-intervention divided by the pooled SD) was used to evaluate the magnitude of the training effect. The level of significance was *p* ≤ 0.05 and SPSS version 20.0 (Somers, NY, United States) software was used.

## Results

There was a statistically significant difference in TTV between the interventions [*F*(1,16) = 20.87, *p* = 0.002 for elbow flexors and *F*(1,16) = 15.98, *p* = 0.004 for elbow extensors]. The elbow flexors TTV during SST-CT was not significantly different (*p* = 1.000) from TRT, while the TTV during the SST-RIV was significantly less than SST-CT (*p* = 0.001) and TRT (*p* = 0.003; Table 1). The elbow extensors TTV during TRT was significantly greater than SST-CT (*p* = 0.023) and SST-RIV (*p* = 0.007). The TTV for the elbow extensors during SST-CT was also significantly greater (*p* = 0.024) than SST-RIV.

**Table 1:** Mean ± SD total training volume (TTV) of elbow flexors, and elbow extensors for sarcoplasma stimulating training contraction type (SST-CT), SST rest interval variable (SST-RIV), and traditional resistance training (TRT) sessions.

Sessions	Elbow flexors	Elbow extensors
SST-CT, AU	1444 ± 397	1957 ± 737
SST-RIV, AU	789 ± 237^∗^	1035 ± 298^∗^
TRT, AU	1531 ± 447^†^	2476 ± 1002^∗†^

*AU, arbitrary units. ^∗^*p* ≤ 0.05 for SST-CT; ^†^*p* ≤ 0.05 for SST-RIV*.

**Figure 1:**
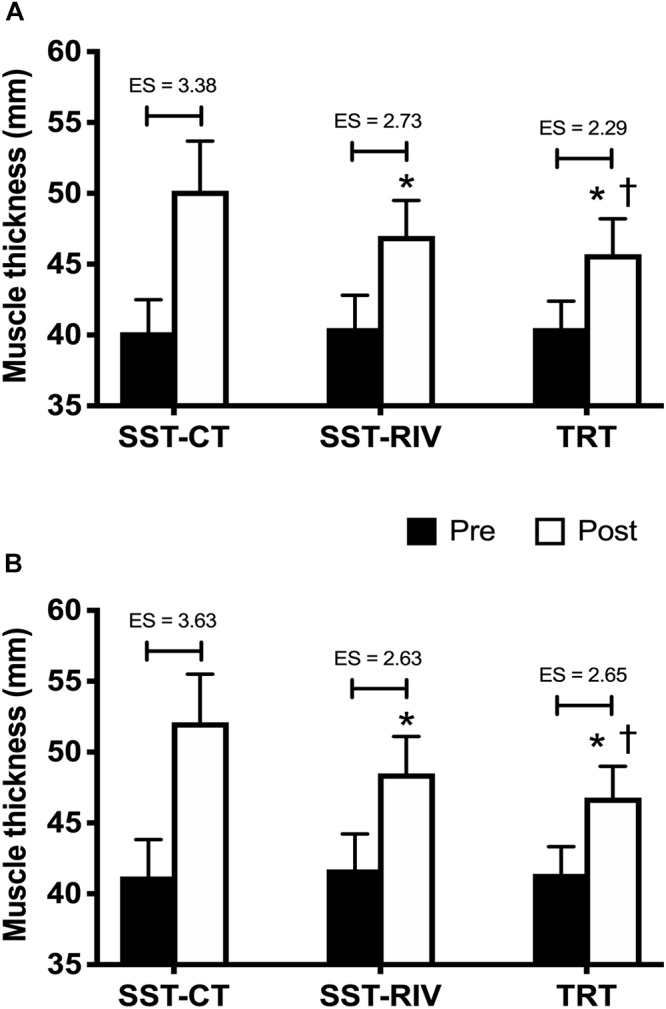
Mean ± SD of biceps brachii (BB) **(A)**, and triceps brachii (TB) **(B)** pre- and post sarcoplasma stimulating training contraction type (SST-CT), SST rest interval variable (SST-RIV), and traditional resistance training (TRT) sessions. ES, effect size; ^∗^*p* ≤ 0.05 for SST-CT after adjustment for pre-intervention; ^†^*p* ≤ 0.05 for SST-RIV after adjustment for pre-intervention.

Arm MT pre- and post SST-CT, SST-RIV, and TRT sessions are shown in Figure 1. After adjustment for pre-intervention MT, there was a statistically significant difference in post-intervention MT between the interventions [*F*(2,26) = 51.41, *p* < 0.0005 for BB, and *F*(2,26) = 91.43, *p* < 0.0005 for TB]. MT presented significant increases (*p* ≤ 0.05) following each intervention as follows: (SST-CT: 10.0 ± 1.3 mm for BB, and 10.9 ± 1.3 mm for TB; SST-RIV: 6.5 ± 0.7 mm for BB, and 6.7 ± 0.7 mm for TB; TRT: 5.1 ± 1.3 mm for BB, and 5.3 ± 0.5 mm for TB). After adjustment for pre-intervention MT, the SST-CT session presented significantly greater increases (*p* ≤ 0.05) in BB and TB MT versus the SST-RIV and TRT sessions. The SST-RIV session also presented significantly greater increases (*p* ≤ 0.05) in BB and TB MT versus the TRT session.

Blood lactate concentration also presented significant increases (*p* ≤ 0.05) following all conditions (Table 2). However, after adjustment for pre-intervention values, there were no significant differences versus post-exercise [*F*(2,26) = 0.48, *p* = 0.627] across training sessions.

**Table 2:** Mean ± SD, percentage of change, and effect size (ES) of blood lactate pre and post sarcoplasma stimulating training contraction type (SST-CT), SST rest interval variable (SST-RIV), and traditional resistance training (TRT) sessions.

Sessions	Pre	Post	Change (Δ)	ES
SST-CT	1.9 ± 0.5	8.4 ± 1.5^∗^	6.5 ± 1.7	5.73
SST-RIV	1.8 ± 0.5	8.3 ± 1.6^∗^	6.5 ± 1.5	5.34
TRT	1.8 ± 0.4	8.5 ± 1.3^∗^	6.7 ± 1.1	7.13

^∗^*p ≤ 0.05 for Pre-intervention*.

## Discussion

The initial hypothesis of the present study was partially confirmed, as the SST sessions resulted in greater acute BB and TB MT versus the traditional RT session, while there was no difference in lactate levels between them. Furthermore, SST-CT also resulted in greater BB and TB MT versus the SST-RIV session. TTV was greater in the traditional RT session versus both SST sessions, except in the case of the elbow flexors, for which the results were not statistically different between the TRT and SST-CT. The SST-CT resulted in greater TTV versus the SST-RIV for both exercises tested. To the best of our knowledge, this is the first paper to report and to compare the acute effects of variations of the SST method versus traditional RT.

Among the possible strategies to increase TTV are the prescription of longer rest intervals, load reductions over consecutive sets for consistency in repetitions, an increase in the number of sets, and exercises that can be used in a variety of RT methods (American College of Sports Medicine, 2009). Furthermore, a higher TTV has been shown to be a key factor associated with muscle hypertrophy (Schoenfeld et al., 2019); albeit, the time under tension, type of muscle action, and exercise mode can also be relevant contributors (American College of Sports Medicine, 2009; Grgic et al., 2018).

For example, Penzer et al. (2016) utilized an elbow flexion movement and compared a 3/7 method consisting of 1 set of 3 repetitions, 1 set of 4 repetitions, 1 set of 5 repetitions, 1 set of 6 repetitions, and 1 set of 7 repetitions with 15 s rest interval between sets with traditional approaches of 4 × 6 and 8 × 6 repetitions with 70% of 1-RM and 150 s rest between sets. The 3/7 method was accompanied by greater muscle activity and tissue oxygenation deficits; thus, very brief rest intervals between sets during elbow curl resulted in greater metabolic demand. These results are somewhat similar to those observed during SST sessions, as the method consists of very short rest interval pauses, and induced higher acute MT than the traditional approach, probably due to a more elevated local metabolic demand. Moreover, Stragier et al. (2019) added more information about the 3/7 and revealed that greater muscle strength and hypertrophy gains was found in the elbow flexors versus a traditional protocol consisting of 8 × 6 with 150 s rest between sets, with training sessions being similar in intensity and volume. This might indicate that mechanisms such as greater acute metabolic stress and muscle pump associated with greater workout density (volume per unit time) may stimulate greater increases in muscle mass and strength (Wackerhage et al., 2019), which might also occur for SST.

Although the SST sessions presented significantly less TTV versus the traditional method, the high local metabolic stress and very short rest interval between sets combined with emphasis on different muscle actions stimulated a more pronounced acute increase in MT, as observed by the ultrasound images, while lactate levels were not statistically different between them. It is possible that local muscle metabolic stress can be higher without significant differences in circulating markers, such as lactate. For example, skeletal muscle is capable of producing local factors following resistance exercise, such as inlerleukin-6 and tumor necrosis factor-alpha, without significant modifications of these biochemical signaling molecules in the blood stream, which alter muscle environment to favor beneficial adaptations to training (Steensberg et al., 2002). Of note is the fact that there is an association of acute muscle pump with the activation of integrin, a membrane protein, responsible for the triggering of intracellular anabolic mechanisms, and the reduction of catabolic processes, accompanied by increased muscle protein synthesis (Wackerhage et al., 2019).

Marshall et al. (2012) examined well-trained subjects in performing the squat exercise at an intensity of 80% 1-RM with 5 sets of 4 repetitions and 3 min rest intervals between sets versus 5 sets of 4 repetitions with 20 s inter-set rest intervals or a rest-pause method with the first set to failure, and subsequent sets performed with a 20 s inter-set rest interval up to 20 repetitions. The rest-pause method resulted in greater electromyographic activity of several lower limb muscles versus the other training sessions. It is very difficult to compare previous studies with our results, as the proposed SST method is widely different from other traditional methods, while it seems that even trained subjects unaccustomed to performing a specific training approach with very short rest intervals will experience higher local metabolic stress, an increase in MT, and possible limited tissue oxygenation, as observed with rest-pause and 3/7 methods.

Total training volume, time under tension, and metabolic stress seem to be important variables to alter acute and chronic physiological responses to RT, albeit it is impossible to determine the most important of them. Valamatos et al. (2018) examined untrained subjects in performing leg extension training for 15 weeks; three sessions/week with partial (0–60° of knee flexion) and full range of motion (ROM) (0–100° of knee flexion), and time under tension equated for both groups. The authors reported that the group performing the partial ROM training displayed joint angle-specific strength gains, with greater increases around the trained joint angles, and also similar gains in muscle hypertrophy. Moreover, during the sessions, torque and total mechanical work were greater in the full ROM group, despite having the same time allocation under tension. In the present study, training sessions had a different TTV, which preserves their practical use, while MT responses were higher for the SST sessions, especially the SST-CT session. These results may confirm the original name and idea of the method proposed by Patrick Tuor, as SST.

Untrained subjects were examined in performing three sets of bilateral 45° leg press, and three sets of bilateral leg extension with 9–12RM, and 90 s rest between sets over 10 weeks, three sessions per week (Damas et al., 2016). The results confirmed that during the first 5 weeks of training the increase in the vastus lateralis cross-sectional area was associated with edema–induced muscle swelling, while this effect was not present after 10 weeks, even with increased muscle hypertrophy. Thus, the results of the present study indicate that the SST methods induced more pronounced effects on BB and TB MT. Moreover, metabolic stress induced by RT involves an increase in intracellular hydration, and the raising of water content of the muscle cells (cell swelling), which has been suggested as an important stimulus for muscle growth in a condition of higher metabolic accumulation (Loenneke et al., 2012; Schoenfeld, 2013). Some limitations of the present study include the lack of measures from muscle activation, muscle oxygenation, and muscle strength before and after the training sessions that could supply valuable information about the physiological stress imposed by the SST method.

## Conclusion

The well-trained characteristic of the studied subjects, the adequate sample power, and the strong control of training variables increase the validity of the present results. Highly trained subjects may benefit from changing their RT routines by using the SST method, as this method may offer a superior MT stimulus, even with a lower TTV versus a more traditional approach. However, the chronic effects of the SST method on muscle hypertrophy and muscle strength remain to be determined. This is the first study ever to offer some interesting physiological insights about SST, a widely used method among trained subjects and bodybuilders. Nevertheless, the question remains if the differences observed between SST approaches will result in distinct chronic adaptations.

## Data Availability

All datasets generated for this study are included in the manuscript and/or the supplementary files.

## Ethics Statement

Methodist University of Piracicaba Research Ethics Committee (Protocol 1.792.429).

## Author Contributions

All authors listed have made a substantial, direct and intellectual contribution to the work, and approved it for publication.

## Conflict of Interest Statement

The authors declare that the research was conducted in the absence of any commercial or financial relationships that could be construed as a potential conflict of interest.

## References

[B1] American College of Sports Medicine (2009). American College of Sports Medicine Position Stand. Progression models in resistance training for healthy adults. *Med Sci Sports Exerc* 41 687–708. 10.1249/MSS.0b013e3181915670 19204579

[B2] Burd N. A. West D. W. Staples A. W. Atherton P. J. Baker J. M. Moore D. R. (2010). Low-load high volume resistance exercise stimulates muscle protein synthesis more than high-load low volume resistance exercise in young men. *PLoS One* 5:e12033. 10.1371/journal.pone.0012033 20711498 PMC2918506

[B3] Damas F. Phillips S. M. Lixandrao M. E. Vechin F. C. Libardi C. A. Roschel H. (2016). Early resistance training-induced increases in muscle cross-sectional area are concomitant with edema-induced muscle swelling. *Eur J Appl Physiol* 116 49–56. 10.1007/s00421-015-3243-4 26280652

[B4] Deschenes M. R. Kraemer W. J. (2002). Performance and physiologic adaptations to resistance training. *Am J Phys Med Rehabil* 81 S3–S16. 12409807 10.1097/00002060-200211001-00003

[B5] Faul F. Erdfelder E. Lang A. G. Buchner A. (2007). G^∗^Power 3: A flexible statistical power analysis program for the social, behavioral, and biomedical sciences. *Behavior Research Methods* 39 175–191. 17695343 10.3758/bf03193146

[B6] Grgic J. Homolak J. Mikulic P. Botella J. Schoenfeld B. J. (2018). Inducing hypertrophic effects of type I skeletal muscle fibers: A hypothetical role of time under load in resistance training aimed at muscular hypertrophy. *Med Hypotheses* 112 40–42. 10.1016/j.mehy.2018.01.012 29447936

[B7] Hanson E. D. Srivatsan S. R. Agrawal S. Menon K. S. Delmonico M. J. Wang M. Q. (2009). Effects of strength training on physical function: influence of power, strength and body composition. *J Strength Cond Res* 23 2627–2637. 19910811 10.1519/JSC.0b013e3181b2297b PMC2966873

[B8] Loenneke J. P. Fahs C. A. Rossow L. M. Abe T. Bemben M. G. (2012). The anabolic benefits of venous blood flow restriction training may be induced by muscle cell swelling. *Med Hypotheses* 78 151–154. 10.1016/j.mehy.2011.10.014 22051111

[B9] Marshall P. W. M. Robbins D. A. Wrightson A. W. Siegler Jason C. (2012). Acute neuromuscular and fatigue responses to the rest-pause method. *J Sci Med Sport* 15 153–158. 10.1016/j.jsams.2011.08.003 21940213

[B10] Paoli A. Moro T. Marcolin G. Neri M. Bianco A. Palma A. (2012). High-Intensity Interval Resistance Training (HIRT) influences resting energy expenditure an respiratory ratio in non-dieting individuals. *J Transl Med* 10 237. 10.1186/1479-5876-10-237 23176325 PMC3551736

[B11] Penzer F. Cabrol A. Baudry S. Duchateau J. (2016). Comparison of muscle activity and tissue oxygenation during strength training protocols that differ by their organization, rest interval between sets, and volume. *Eur J Appl Physiol* 116 1795–1806. 10.1007/s00421-016-3433-8 27439989

[B12] Prestes J. Foschini D. Marchetti P. H. Charro M. A. Tibana R. A. (2016). *Prescrição e periodização do treinamento de força em academias*, 2nd Edn. Barueri, SP: Manole.

[B13] Prestes J. Nascimento D. C. Tibana R. A. Teixeira T. G. Vieira D. C. L. Farias D. L. (2015). Undertanding the individual reponsiveness to resistance training periodization. *AGE* 37 37–55. 10.1007/s11357-015-9793-x 25971877 PMC4430497

[B14] Prestes J. Tibana R. A. de Araujo Sousa E. da Cunha Nascimento D. de Oliveira Rocha P. Camarço N. F. (2017). Strength And Muscular Adaptations Following 6 Weeks Of Rest-Pause Versus Traditional Multiple-Sets Resistance Training In Trained Subjects. *J Strength Cond Res* 10.1519/JSC.0000000000001923 [Epub ahead of print]. 28617715

[B15] Schoenfeld B. J. (2013). Potential mechanisms for a role of metabolic stress in hypertrophic adaptations to resistance training. *Sports Med* 43 179–194. 10.1007/s40279-013-0017-1 23338987

[B16] Schoenfeld B. J. Contreras B. Krieger J. Grgic J. Delcastillo K. Belliard R. (2019). Resistance Training Volume Enhances Muscle Hypertrophy. *Med Sci Sports Exerc* 51 94–103. 10.1249/MSS.0000000000001764 30153194 PMC6303131

[B17] Schoenfeld B. J. Ogborn D. Krieger J. W. (2017). Dose response relationship between weekly resistance training volume and increases in muscle mass: A systematic review and meta-analysis. *J Sports Sci* 35 1073–1082. 10.1080/02640414.2016.1210197 27433992

[B18] Steensberg A. Keller C. Starkie R. L. Osada T. Febbraio M. A. Pedersen B. K. (2002). IL-6 and TNF-α expression in, and release from, contracting human skeletal muscle. *J Physiol Endocrinol Metab* 283 E1272–E1278. 10.1152/ajpendo.00255.2002 12388119

[B19] Stragier S. Baudry S. Carpentier A. Duchateau J. (2019). Efficacy of a new strength training design: the 3/7 method. *Eur J Appl Physiol* 119 1093–1104. 10.1007/s00421-019-04099-5 30756168

[B20] Valamatos M. J. Tavares F. Santo R. M. Veloso A. P. Mil-Homens P. (2018). Influence of full range of motion vs. *equalized partial range of motion training on muscle architecture and mechanical properties*. *Eur J Appl Physiol* 118 1969–1983. 29982844 10.1007/s00421-018-3932-x

[B21] Wackerhage H. Schoenfeld B. J. Hamilton D. L. Lehti M. Hulmi J. J. (2019). Stimuli and sensors that initiate skeletal muscle hypertrophy following resistance exercise. *J Appl Physiol* 126 30–43. 10.1152/japplphysiol.00685.2018 30335577

